# Technology-Enabled Remote Monitoring and Self-Management — Vision for Patient Empowerment Following Cardiac and Vascular Surgery: User Testing and Randomized Controlled Trial Protocol

**DOI:** 10.2196/resprot.5763

**Published:** 2016-08-01

**Authors:** Michael McGillion, Jennifer Yost, Andrew Turner, Duane Bender, Ted Scott, Sandra Carroll, Paul Ritvo, Elizabeth Peter, Andre Lamy, Gill Furze, Kirsten Krull, Valerie Dunlop, Amber Good, Nazari Dvirnik, Debbie Bedini, Frank Naus, Shirley Pettit, Shaunattonie Henry, Christine Probst, Joseph Mills, Elaine Gossage, Irene Travale, Janine Duquette, Christy Taberner, Sanjeev Bhavnani, James S Khan, David Cowan, Eric Romeril, John Lee, Tracey Colella, Manon Choinière, Jason Busse, Joel Katz, J Charles Victor, Jeffrey Hoch, Wanrudee Isaranuwatchai, Sharon Kaasalainen, Salima Ladak, Sheila O'Keefe-McCarthy, Monica Parry, Daniel I Sessler, Michael Stacey, Bonnie Stevens, Robyn Stremler, Lehana Thabane, Judy Watt-Watson, Richard Whitlock, Joy C MacDermid, Marit Leegaard, Robert McKelvie, Michael Hillmer, Lynn Cooper, Gavin Arthur, Krista Sider, Susan Oliver, Karen Boyajian, Mark Farrow, Chris Lawton, Darryl Gamble, Jake Walsh, Mark Field, Sandra LeFort, Wendy Clyne, Maria Ricupero, Laurie Poole, Karsten Russell-Wood, Michael Weber, Jolene McNeil, Robyn Alpert, Sarah Sharpe, Sue Bhella, David Mohajer, Sem Ponnambalam, Naeem Lakhani, Rabia Khan, Peter Liu, PJ Devereaux

**Affiliations:** ^1^ McMaster University Hamiltion, ON Canada; ^2^ Population Health Research Institute Hamilton, ON Canada; ^3^ Coventry University Coventry United Kingdom; ^4^ Mohawk College Hamilton, ON Canada; ^5^ York University Toronto, ON Canada; ^6^ University of Toronto Toronto, ON Canada; ^7^ Hamilton Health Sciences Hamilton, ON Canada; ^8^ Liverpool Heart and Chest Hospital Liverpool United Kingdom; ^9^ Scripps Clinic and Research Institute La Jolla, CA United States; ^10^ University Health Network Toronto, ON Canada; ^11^ University of Montreal Montreal, QC Canada; ^12^ St. Michael's Hospital Toronto, ON Canada; ^13^ Brock University St. Catharines, ON Canada; ^14^ Cleveland Clinic Cleveland, OH United States; ^15^ Oslo and Akershus University College of Applied Sciences Oslo Norway; ^16^ Ministry of Health and Long-Term Care Toronto, ON Canada; ^17^ Canadian Pain Coalition Toronto, ON Canada; ^18^ Heart and Stroke Foundation Ottawa, ON Canada; ^19^ Canadian Cardiovascular Society Ottawa, ON Canada; ^20^ Memorial University Of Newfoundland St. Johns, NL Canada; ^21^ Ontario Telemedicine Network Toronto, ON Canada; ^22^ Philips Toronto, ON Canada; ^23^ QoC Health Toronto, ON Canada; ^24^ XAHIVE Toronto, ON Canada; ^25^ mPath Toronto, ON Canada; ^26^ University of Ottawa Heart Institute Ottawa, ON Canada

**Keywords:** technology-enabled self-management, remote automated external monitoring, usability testing, randomized controlled trial

## Abstract

**Background:**

Tens of thousands of cardiac and vascular surgeries (CaVS) are performed on seniors in Canada and the United Kingdom each year to improve survival, relieve disease symptoms, and improve health-related quality of life (HRQL). However, chronic postsurgical pain (CPSP), undetected or delayed detection of hemodynamic compromise, complications, and related poor functional status are major problems for substantial numbers of patients during the recovery process. To tackle this problem, we aim to refine and test the effectiveness of an eHealth-enabled service delivery intervention, TecHnology-Enabled remote monitoring and Self-MAnagemenT—VIsion for patient EmpoWerment following Cardiac and VasculaR surgery (THE SMArTVIEW, CoVeRed), which combines remote monitoring, education, and self-management training to optimize recovery outcomes and experience of seniors undergoing CaVS in Canada and the United Kingdom.

**Objective:**

Our objectives are to (1) refine SMArTVIEW via high-fidelity user testing and (2) examine the effectiveness of SMArTVIEW via a randomized controlled trial (RCT).

**Methods:**

CaVS patients and clinicians will engage in two cycles of focus groups and usability testing at each site; feedback will be elicited about expectations and experience of SMArTVIEW, in context. The data will be used to refine the SMArTVIEW eHealth delivery program. Upon transfer to the surgical ward (ie, post-intensive care unit [ICU]), 256 CaVS patients will be reassessed postoperatively and randomly allocated via an interactive Web randomization system to the intervention group or usual care. The SMArTVIEW intervention will run from surgical ward day 2 until 8 weeks following surgery. Outcome assessments will occur on postoperative day 30; at week 8; and at 3, 6, 9, and 12 months. The primary outcome is worst postop pain intensity upon movement in the previous 24 hours (Brief Pain Inventory-Short Form), averaged across the previous 14 days. Secondary outcomes include a composite of postoperative complications related to hemodynamic compromise—death, myocardial infarction, and nonfatal stroke— all-cause mortality and surgical site infections, functional status (Medical Outcomes Study Short Form-12), depressive symptoms (Geriatric Depression Scale), health service utilization-related costs (health service utilization data from the Institute for Clinical Evaluative Sciences data repository), and patient-level cost of recovery (Ambulatory Home Care Record). A linear mixed model will be used to assess the effects of the intervention on the primary outcome, with an a priori contrast of weekly average worst pain intensity upon movement to evaluate the primary endpoint of pain at 8 weeks postoperation. We will also examine the incremental cost of the intervention compared to usual care using a regression model to estimate the difference in expected health care costs between groups.

**Results:**

Study start-up is underway and usability testing is scheduled to begin in the fall of 2016.

**Conclusions:**

Given our experience, dedicated industry partners, and related RCT infrastructure, we are confident we can make a lasting contribution to improving the care of seniors who undergo CaVS.

## Introduction

### Background

Cardiac and vascular surgeries (CaVS) are performed on seniors [1] to improve survival and health-related quality of life (HRQL). Unfortunately, chronic postsurgical pain (CPSP), delayed detections of hemodynamic compromise, complications, and related poor functional status are major problems for substantial numbers of recovering patients [1]. This reflects the inadequacy of current systems for patient monitoring after CaVS, both on hospital surgical wards and at home. The current approach (eg, manually checking vital signs every 8-12 hours on postsurgical wards) results in thousands of cases of delayed detection of hemodynamic compromise (eg, low blood pressure and hypoxia) leading to severe complications (eg, myocardial infarction and stroke) [2] and drastically reduced HRQL. To tackle this problem, our aim is to refine and test the effectiveness of the eHealth-enabled service delivery intervention, TecHnology-Enabled remote monitoring and Self-MAnagemenT—VIsion for patient EmpoWerment following Cardiac and VasculaR surgery (THE SMArTVIEW CoVeRed), which combines remote automated monitoring, education, and self-management training to optimize recovery in seniors undergoing CaVS, internationally.

### Population, Challenges, Gaps, and Inefficiencies to be Addressed

#### Overview

Collectively as an innovation community we have completed, or are conducting, prospective outcome studies with >65,000 surgical patients, including CaVS patients [3-12]. Based upon the collective research and literature syntheses [13-15], CaVS can be currently characterized by several clinical inefficiencies, resulting in the key challenges discussed in the following sections.

#### Chronic Postsurgical Pain and Related Consequences

CaVS surgeries affect pain-sensitive structures as they invade muscle and visceral tissues, and involve the harvesting and manipulation of vessels. Such surgical tissue insults lead to pathological nervous system changes, collectively known as sensitization [16]—a function of neuronal modifiability [17]. Sensitization increases pain sensitivity (ie, hyperalgesia), augments the normal duration (ie, hyperpathia) and amplitude of pain, and results in abnormal interpretation of nonpainful stimuli as painful (ie, allodynia) [16]. In all cases, CPSP is, in part, a function of unrelieved acute postoperative (postop) pain that involves a transition phase [18] by virtue of these pathological mechanisms. In our review of 26 studies (n=2033; mean age 65.1 years) across 15 countries, CPSP prevalence following CaVS [11,12,18-41] ranged from 17-56%. The 2013 Canadian prospective Cardiac (CARD) pain study (n=1010) [11] reported more modestly varying CPSP prevalence rates of 40%, 22%, and 17% at 3, 6, and 12 months following surgery, respectively; pain was most commonly located along the sternal incision and saphenous vein harvesting sites. But other studies have reported 1-year CPSP prevalence rates as high as 39% [39] and 45% [40]. Rates of CPSP following vascular surgery are similar in range (25%), with moderate to severe pain typically presenting along the femoropopliteal bypass tunnel [42].

The deleterious consequences of CPSP in CaVS—amidst divergent surgical populations—are well-known, with numerous studies reporting associations of CPSP with poor HRQL and depressive disorder [11,12,18-43]. We meta-analyzed available data [11,25,26,29,32,33,35] (see Multimedia Appendix 1) and found that among seniors who undergo CaVS, there is a statistically and clinically significant relationship between acute postop pain and CPSP development (standardized mean difference 0.28; 95% CI 0.12-0.44) (see Table 1). This emphasizes the decades of research [44-53] which indicate that CaVS patients have erroneous pain and pain medication beliefs that obstruct acute postop pain management. In 2004, Watt-Watson et al [46] found that up to 83% of CaVS patients do not ask for pain medication when requiring it and that, on average, <35% of prescribed analgesic dosages are routinely administered [46]. Current studies indicate that this unfortunate scenario remains unchanged. Cogan et al [53] recently found, for example, that 36% of CaVS patients believed that “pain medication should be spared until the pain is very severe” and 20% believed that “good patients do not speak of their pain.” A gap revealed from this meta-analysis regarding CPSP was that unrelieved acute postop pain requires more effective intervention. A solution to this gap is that postop education, support, and acute pain monitoring and management at home are needed to prevent transition from acute postop pain to CPSP.

**Table 1:** Meta-analysis: Differences in acute postoperative pain scores between those who do and do not develop chronic postsurgical pain.

Study	Standardized mean difference (SE)	Weight (%)	Standardized mean difference inverse variance random effects (95% CI)
Choiniere et al 2014 [11]	0.14 (0.04)	24.2	0.14 (0.06 to 0.22)
King et al 2008 [25]	0.07 (0.11)	17.3	0.07 (-0.15 to 0.29)
Lahtinen et al 2006 [26]	0.13 (0.06)	22.7	0.13 (0.02 to 0.25)
Lee et al 2010 [35]	0.96 (0.39)	3.8	0.96 (0.19 to 1.74)
Steegers et al 2007 [29]	0.86 (0.17)	12.3	0.86 (0.53 to 1.19)
van Gulik et al 2011 [33]	0.31 (0.17)	12.6	0.31 (-0.02 to 0.63)
van Gulik et al 2012 [32]	0.27 (0.25)	7.1	0.27 (-0.25 to 0.79)
Total	N/A^a^	N/A	0.28 (0.12 to 0.44)

^a^N/A: not applicable.

#### Undetected Hemodynamic Compromise

CaVS are among the highest-risk surgeries and are associated with substantial postop morbidity and mortality. Following an immediate postop period of intensive care unit (ICU) hemodynamic surveillance, vital signs monitoring after ICU discharge is lacking. Most patients on surgical wards will have vital signs evaluated once per 4-12 hours [54,55]. Such limited in-hospital monitoring—followed by no daily monitoring at home—is significantly associated with poor clinical outcomes. For example, in a study from the Cleveland Clinic [56], nurses blinded to continuous pulse oximetry for monitoring peripheral oxygen saturation (SpO_2_) assessed their postop patients (n=594) according to normal practice and detected a 5% incidence of hypoxemia (SpO_2_< 90%). Blindly captured study oximetry, however, detected that 37% of patients had one or more continuous episodes of hypoxemia for ≥1 hour, and that 10% of patients had at least one continuous episode (≥1 hour) of hypoxemia where SpO_2_ was <85% [56]. Given that hypoxemia for >5 minutes is associated with increased risk of myocardial ischemia, suboptimal monitoring on surgical wards elevates risks for patients. Studies have also demonstrated that continuous electrocardiographic ST segment monitoring after surgery can identify asymptomatic ischemia that is independently associated with myocardial infarction [57-59]. A study of postop ST segment depression followed 151 consecutive patients undergoing major vascular surgery and assessed for postop myocardial ischemia [57]. Approximately 85% of patients who suffered postop cardiac events had preceding long-duration ST segment depressions [57]. These data suggest that remote, continuous, noninvasive ST segment monitoring systems can identify impending cardiac events much sooner than the usual practice of checking vital signs manually every 4-12 hours. The same is true for cardiac postop arrhythmias. The incidence of atrial fibrillation, in particular, is 20-40% after cardiac surgery, with even higher rates (30-50%) after valvular surgery [60-62]. While most patients spend the first 12-24 hours after surgery in the ICU, 70% of postop atrial fibrillation occurs over the first 4 days following surgery, suggesting that many occurrences will be missed on surgical wards [63]. This common scenario is risk elevating, given that atrial fibrillation imposes a three-fold increase in hypotension and stroke [64,65].

Data from large randomized controlled trials (RCTs) also suggest that blood pressure is a particularly important independent predictor of postop cardiac complications and death. The PeriOperative ISchemic Evaluation (POISE) trial [4] randomized 8351 patients to extended-release metoprolol (mean age 68.9 years) or placebo (mean age 69.1 years). Along with a reduction in myocardial infarction, a clinically significant increase in hypotension with metoprolol use was found (hazard ratio [HR] 1.55; 95% CI 1.38-1.74). Overall, clinically significant hypotension is associated with the largest population-attributable risk for perioperative death and perioperative stroke [4]. Following POISE, POISE-2 was an international RCT of 10,010 patients with, or at risk of, vascular disease undergoing noncardiac surgery, including vascular surgery [5]. Analyses demonstrated that clinically important hypotension was an independent predictor of subsequent risk of myocardial infarction during 30-day follow-up (adjusted HR 1.37; 95% CI 1.16-1.62) [5]. A gap revealed from this analysis was that current monitoring of patients after CaVS is inadequate, with significant harm resulting from undetected postoperative hypoxemia, arrhythmia, and hypotension. A solution to this gap is remote automated noninvasive postoperative monitoring, for 30 days following discharge, to enhance detection of hemodynamic compromise and reduce adverse event risk.

#### Surgical Site Infections

CaVS options are changing, with many patients choosing percutaneous coronary interventions (PCIs) to address vasculature blockages. This results in those undergoing CaVS manifesting disease that is either too advanced or too complicated for PCI. As such, CaVS patients—often with multiple comorbidities—are at high risk for surgical site infections (SSIs). In England, for example, SSIs occurred in 4.4% of patients (n=29,144) who underwent coronary artery bypass grafting and 2.2% of patients (n=7256) who underwent vascular surgery—in National Health Service hospitals from April 2008 to March 2013 [66]. The median time to infection identification was 12 days and 11 days after cardiac and vascular surgery, respectively [66]. A recent systematic review—57 studies—has corroborated the commonality of these infection rates and that SSIs, furthermore, have major consequences including mortality, repeated surgical procedures, hospital readmissions, and health-related economic burden [67]. Evidence from established daily postoperative surveillance systems in the United States suggests that daily wound monitoring can prevent SSI progression—superficial/incisional to deep wound/organ/space [68]. A gap revealed from this analysis is that postop SSIs often manifest at home following discharge and are potentially preventable [69]. A solution to this gap would be daily postop wound monitoring for early detection of, and to prevent progression of, SSIs that require hospitalization.

### Hospital Readmissions and Summary of Key Issues

Not only are CaVS among the highest-risk surgeries, they are associated with high rates of hospital readmission. A 2014 prospective, multicenter cohort study—10 centers, 5185 patients—in Canada and the United States reported the rate of all-cause 30-day readmission following cardiac surgery at 18.7% [69]. Recent data (2014) from a large US registry (N=11,246) showed comparable rates of 30-day unplanned readmissions among major vascular surgery patients: 15.7%, infrainguinal bypass [70]. Our particular focus on the aforementioned issues is due to the unequivocal association with poor postoperative functional recovery resulting in CPSP [11,12,18-41]; adverse cardiac events due to hemodynamic compromise [54-59]; and high rates of hospital readmission due to infection [66,69]. Postoperative infection, for example, is the most common reason for readmission in Canada following cardiac surgery (17.1%) [69]. Other important recovery challenges for seniors, requiring intervention, include psychological morbidity (eg, anxiety and depression) [71-77] and medication reconciliation [78].

### Proposed eHealth Innovation-Enabled Care Delivery Program

#### Intervention in Canada and the United Kingdom

This project is being undertaken in Canada and the United Kingdom because (1) the gaps and inefficiencies following CaVS are similar and (2) implementations of eHealth innovations require attention to agile/scalable designs which can be realized through efficient (ie, parallel) integration and effectiveness testing across two health systems.

#### Targeting Seniors Recovering From Cardiac and Vascular Surgery

Guided by the Integrated Vascular Health Blueprint for Ontario [79] and the UK Department of Health Cardiovascular Disease Outcomes Strategy [80], our aim is health service integration. The fragmented nature of cardiac/vascular care threatens the sustainability of health systems due to inefficiencies and waste. Given that factors associated with poor recovery are common to both sets of CaVS patients, we are aligned with the Canadian Vascular Health Coalition strategy—see page 7 of the Integrated Vascular Health Blueprint for Ontario [79]—of mapping and implementing more integrated ways of addressing cardiac and vascular disease-related burden.

#### Partnership Process and Technology Partners

Initial discussions have centered on eHealth Innovation Partnership Program objectives, CaVS recovery challenges, and potential partners’ orientation to improving patient experience, willingness to codesign, and their match with desired partner criteria. Further discussions reviewed respective technology innovations and desired scope of involvement. As a result of this process, we are fortunate to be working with Philips Canada, QoC Health, XAHIVE, and mPath. These partners are drivers of innovation, ranging from small to medium enterprises, to a multinational organization.

#### Guiding Principles, Work to Date, and Patient-Oriented Approach

The intervention has been designed according to *Patients First: Action Plan for Health Care* [81]. Grounded in commitment to efficiency and integration of care, the following tenets of Patients First serve as our guiding principles: (1) Improve access: provide faster access to the right care by removing barriers to full scope of practice and coordinating care, (2) Connect patients to services: deliver integrated care that is home based when possible, (3) Protect public health care system: innovate based on evidence and capacity to engage patients, and (4) Inform: provide education and transparency. With Patients First as our framework, our leadership team and technology partners jointly applied for, and secured, competitive seed funding from the Michael G DeGroote Institute for Pain Research and Care at McMaster University. With these funds, a 2-day, international SMArTVIEW meeting was held for the purposes of intervention codesign, systems integration planning, and change management/scalability plan development. A professional facilitation company, Guiding Star Communications, led us through structured patient journey mapping and analysis. Divided into working groups—each with scientists; clinicians; CaVS patient representatives; engineers; and information technology, policy, and knowledge translation experts—we worked from stems of real CaVS cases to map the typical senior patient’s recovery journey, based on experience. We then engaged in facilitated analysis of what “must change.” Once “must change” items were distilled and validated by patient representatives, the technology partners showcased their evidence-based innovations for change. Using consensus techniques, we mapped partners’ solutions to “must change” items in a codesign of the SMArTVIEW intervention. Three subgroups worked intensively on systems integration.

#### Intervention Program, Technologies, and Effectiveness

##### Overview

**Figure 1:**
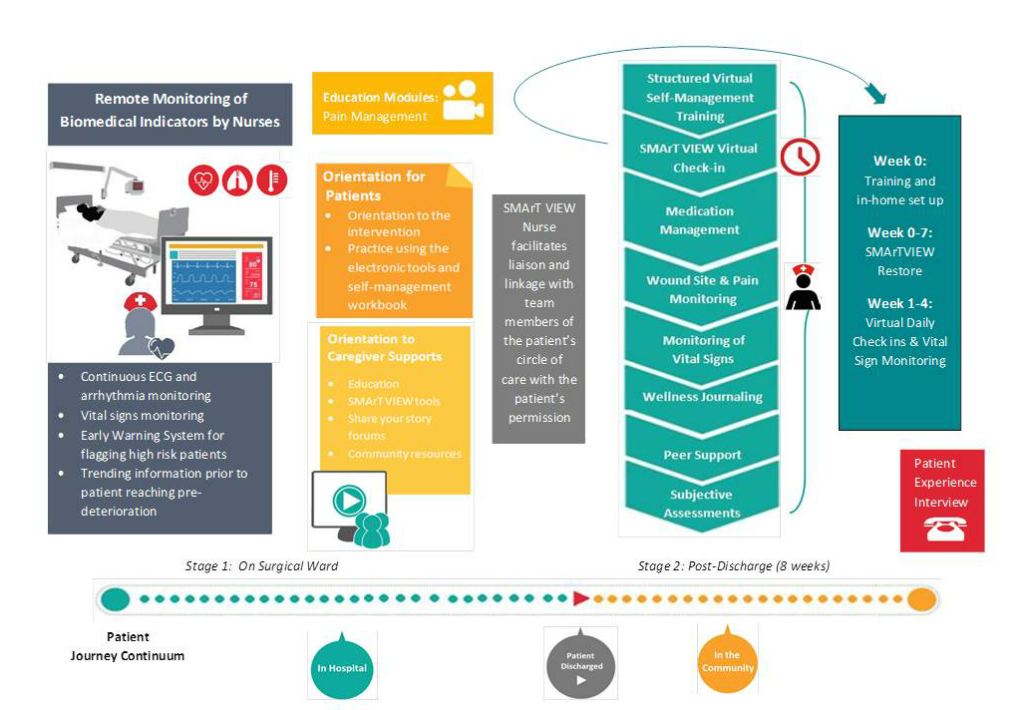
The Self-MAnagemenT—VIsion for patient EmpoWerment (SMArTVIEW) eHealth-enabled service delivery program. ECG: electrocardiogram.

SMArTVIEW is an eHealth-enabled service delivery program—based on existing implementable technology and validated interventions—which combines remote monitoring, education, and self-management training (see Figure 1). The following review of SMArTVIEW components as a whole identifies key members of the health care team as well as key phases and technology enablers.

##### Health Professionals Involved, Phases, and Technology Enablers

Multiple clinicians are involved in seniors’ circles of care in the hospital and the community. Successful implementation, however, requires centralized coordination. Therefore, the “SMArTVIEW Nurse” (SVN), a registered nurse with SVN training, is central. SMArTVIEW is a two-stage intervention program. Stage 1 supports seniors after CaVS in hospital on surgical wards post-ICU, with a view to seamless transition, while Stage 2 supports patients at home during the first 8 weeks of recovery (see Figure 1). Across stages, our clinical technology enablers include Philips’ IntelliVue Guardian [82] and Transition to Ambulatory Care (eTrAC) Program [83], and QoC’s Engagement Platform [84].

##### Stage 1

Stage 1 includes remote automated postoperative monitoring (Protect) and pain management education (Protect, Inform).

###### Monitoring

On the ward, remote monitoring will be implemented by the SVN via Philips’ IntelliVue Guardian early warning system, which includes a centrally located monitor, a portable spot check monitor, and four lightweight cableless devices worn by the patient, with connectivity via short range radio and hotspot transmitters. The four devices are as follows: (1) MX40,a telemetry pack for 8-lead continuous electrocardiogram monitoring; (2) Acquire SpO_2_, a wrist-worn device applied to the index finger, which provides continuous SpO_2_ saturation values under various artifact conditions, including motion and low perfusion, as well as pulse rate; (3) Acquire Blood Pressure, a noninvasive blood pressure cuff worn on the brachial aspect of the arm; and (4) Acquire Respiration Pod, a small patch-like device, attached to the left costal arch of the patient’s chest, which derives respiration rate and patient posture via 3D accelerometer [82]. Receiving data from each device, IntelliVue Guardian software employs a deterioration notification algorithm to facilitate early intervention. This algorithm automates hospital early warning score (EWS) systems, normally performed manually by clinicians. EWSs track vital sign deviations from normal and trigger increasing attention to care, proportional to the deviation. By virtue of automation, IntelliVue Guardian efficiently verifies the accuracy of vital signs data by repeating measurements at customized intervals [82]. If early signs of deterioration are detected, IntelliVue Guardian will inform the SVN via mobile device. Moreover, clinicians on the ward are able to visualize EWS on the central monitor and spot check monitor, which is kept at the patient's bedside. As identified previously, remote automated monitoring is needed to identify undetected hemodynamic compromise and allow for early intervention to prevent adverse events following CaVS; IntelliVue Guardian provides a comprehensive, evidence-based solution [85] to meet this need.

###### Education

Education (Inform) is critical to prevent transition from acute postop pain to CPSP. To empower seniors to know how to communicate their postop pain experience and understand options for pain management (Protect), we employ, on ward day 2, Watt-Watson et al’s Pain Relief After Surgery educational intervention [46]. Adapted as an animated video module, Pain Relief After Surgery is a 20-minute education tool, validated for CaVS patients—comprehension level: Grade 6—and designed to address common misbeliefs preventing patients from asking for improved pain relief. Content also emphasizes the individuality of pain responses and the importance of good pain relief for optimal recovery at home. RCT evidence supports the effectiveness of Pain Relief After Surgery for reducing pain-related interference during recovery as well as misbeliefs about analgesics [46]. In conjunction with Philips' eTrAC program, this video module will be issued to the patient on ward day 2.

##### Stage 2

Stage 2 includes SVN hospital-to-home remote monitoring and support and self-management training.

###### Hospital-to-Home Remote Monitoring and Support

The eTrAC program is a tablet-based solution that combines clinical software for effective care management with Bluetooth-enabled, patient-monitoring devices measuring SpO_2_, blood pressure, temperature, blood glucose, and weight [83]. Philips eTrAC allows clinicians to monitor discharged (ie, at-home) patients’ vital signs status from the hospital (Protect), and then prioritize them for required interventions (eg, signs of sepsis evident) based upon a combination of customized/standardized intervention rules [83]. eTrAC also features customizable, interactive patient symptom and self-report surveys to inform postoperative support and management. SMArTVIEW-specific surveys include postop daily symptoms, wound monitoring, sleep, nutrition, medication, quality of life, and patient satisfaction. Interactive modules assisting patient orientation to the system are also included. The clinician interface for the eTrAC program is eCare Coordinator (eCC), a cloud-based software tool designed to maximize efficiency through risk prioritization— patients are assigned overall scores calculated from weighted scores of self-report surveys, measurements, issues, risk of readmission, and discharge date if within the last 30 days. The overall score generated allows clinicians to manage large patient populations by triaging their interventions based on potential patient need (ie, those with the highest scores are seen first). Through eCC, clinicians can remotely manage patients, view and interpret results (eg, vital signs and symptom and reflexive surveys), follow up and intervene as needed, conduct video visits with patients, and document all patient interventions and observations.

The SVN will employ the eTrAC program to facilitate daily virtual check-ins and counseling, daily vital signs monitoring and triage, and review of interactive symptom and reflexive surveys (Access, Inform, Protect, Connect) [83].

###### Self-Management Training

As with Stage 1, we are committed in Stage 2, combining improved monitoring with education and support that empowers seniors to proactively prevent transition to CPSP and prevent poor functional recovery. As a team we are experienced in the development/testing of self-management models for people with coronary artery disease [86-88], other forms of chronic illness [89,90], and CPSP [91]. In conjunction with postoperative monitoring and support, participants will engage in SMArTVIEW Restore. Restore is a virtual self-management program, to be delivered in 2-hour sessions weekly, over a 7-week period, from week 0 (week of discharge) to week 6 postoperation (at home).

**Figure 2:**
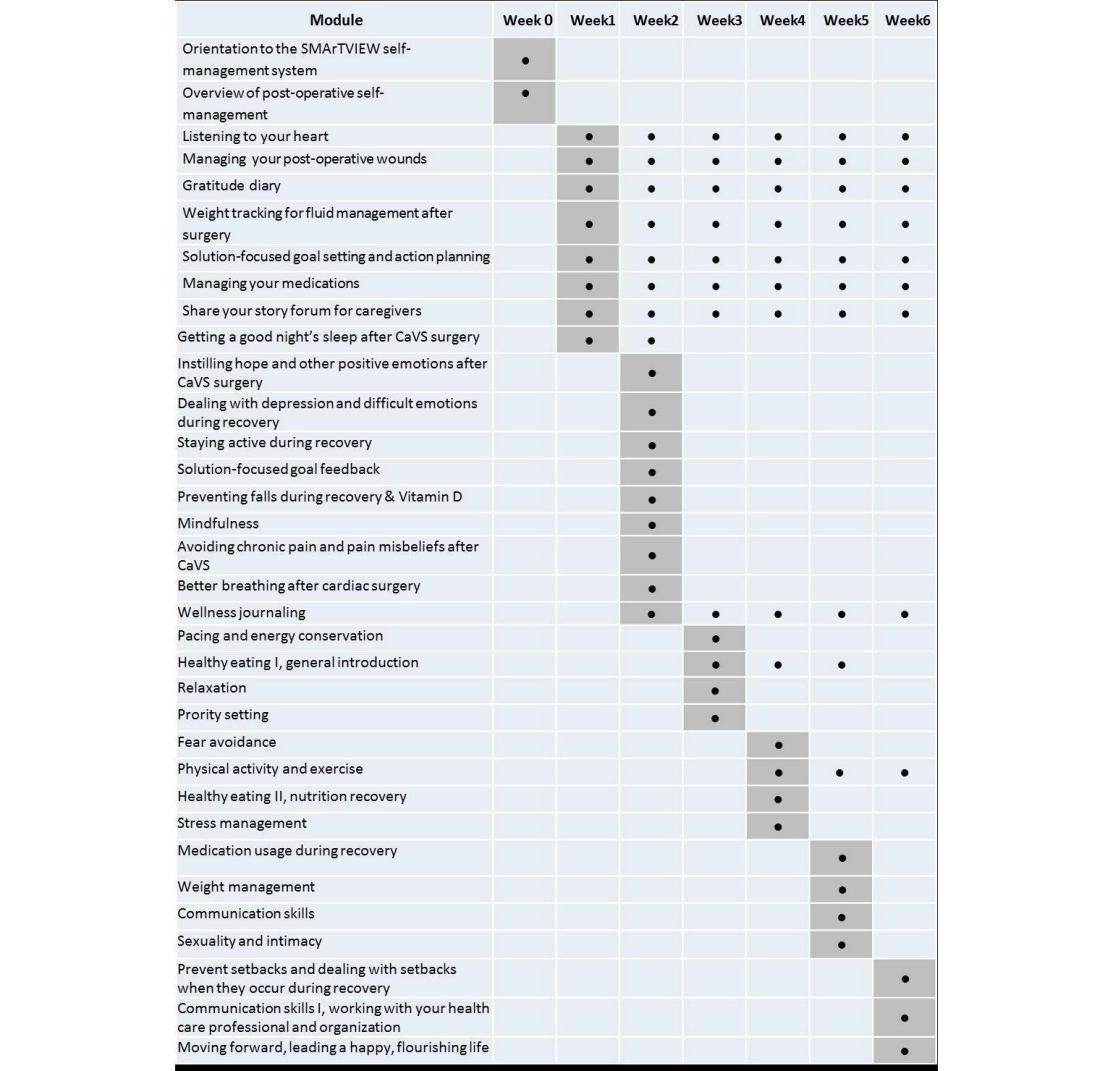
The Self-MAnagemenT—VIsion for patient EmpoWerment (SMArTVIEW) Restore curriculum. CaVS: cardiac and vascular surgery.

Figure 2 presents an overview of the Restore curriculum, based on seniors’ CaVS recovery needs identified during patient journey mapping, as well as lessons learned from our previous self-management experience [86-91]. Both content and process elements of Restore are grounded in the fear-avoidance beliefs model [92], which shows how catastrophic pain perceptions can lead to fear, hypervigilance, avoidance, disability, and depression. The curriculum is designed to provide patients with requisite cognitive, emotional, and behavioral skills to manage their postop pain experience in a productive and positive way, leading to optimal functioning. Restore will be run on a time-release basis; interactive features will include weekly recovery goal setting, interactive reflexive activities, wellness journaling, a peer support forum, and a gratitude “wall.”

Both the online interactive elements as well as self-efficacy-enhancing features of Restore will be adapted specifically from the Coventry University Help to Overcome Problems Effectively (HOPE) and Internet-based (iHOPE) programs. As one of the first self-management interventions combining positive psychology and cognitive behavioral therapy theory, iHOPE includes evidence-based and positive psychological activities such as goal setting, action planning, identifying personal strengths, scheduling pleasant activities, mindfulness, relaxation training, and reviewing successes [93]. Feasibility trials have shown that HOPE has the potential to improve important quality-of-life outcomes for people living with and affected by a range of long-term conditions [94,95]. Recently, Coventry University and Macmillan Cancer Support—the United Kingdom’s leading charity and source of cancer support—tested a Web-based version of HOPE (iHOPE) for cancer survivors, comprised of six interactive Web-based sessions, which combine cancer self-management information and education with self-monitoring tools, worksheets, audio- and video-based materials, interactive goal setting and gratitude “walls,” and social networking via email and discussion forums. Feasibility trial results showed that participants’ depression, anxiety, fatigue, fear of cancer returning, positive mental well-being, hope, and gratitude all significantly improved [95]. Participants’ course experience and usability ratings were high, with all of the participants willing to recommend iHOPE to other users [95].

To facilitate our adaptation of iHOPE interactive elements, QoC Health’s Engagement Platform will be leveraged to customize and integrate the validated modules (ie, feature sets) from their existing platforms and to develop customized modules to transform Restore from concept to a codesigned interactive digital solution. QoC will apply the principles of user interface and user experience design to create a user-friendly and intuitive solution with reduced interface friction. An iterative, user-centered design framework featuring participatory design will be used to develop the Web-based solution, which will be optimized for tablet. QoC will facilitate codesign development sessions with our patient representatives to ensure Restore is aligned with their recovery needs and that it considers their technical capabilities (eg, digital literacy and technology-savvy level) and design preferences. The cognitive load on users will be minimized by abating unnecessary decisions/steps and inconsistencies in the interface. To offer the end user an enhanced e-learning experience throughout Restore, the design will feature “digital resting spaces.” This will be achieved by applying the concepts of e-learning (eg, pacing and quantity and diversity of content) and using the principles of white space to balance content and segregate sections.

In summary, our technology partners are cutting-edge eHealth innovators for change with evidence-based solutions. For example, Philips’ IntelliVue Guardian has been shown to significantly increase timely clinical response in hospital, based on abnormal vital signs detection, as well as survival after rapid response treatment [85]. Emerging evidence also suggests that solutions developed through QoC’s Engagement Platform are feasible, acceptable, and beneficial to postop patients and surgical teams [96,97].

### Systems Integration

SMArTVIEW deploys a highly integrated “system of systems.” Multiple decentralized and heterogeneous subsystems, with operational and managerial independence, are required to provide our end-to-end SMArTVIEW solution. Our end goal for clinical data management is to ensure that the right information is provided to the right person, at the right time. We have consulted extensively with our information technology and clinical informatics experts to leverage existing assets through systems architecture, as opposed to duplicating functionality or existing data. Moreover, our technology partners’ solutions meet Health Level 7 [98] industry standards for seamless connection and bidirectional data exchange with our hospital information and electronic medical record systems. We will also use application programming interfaces for the extraction of data. We are confident we can achieve integrated exchange of information, from hospital to home.

### Privacy and Data Aggregation

Our additional partners, XAHIVE and mPath, serve as our chief stewards of privacy and data aggregation, respectively. With privacy paramount, we espouse a “privacy by design” approach [99]. Privacy will be role based, highly configurable, and will include the entire circle of care, including formal and informal caregivers and supports by patient consent. To achieve these objectives, we will employ XAHIVE’s secure communication service platform, extensible using a custom off-the-shelf model. The XAHIVE communication protocol does not require servers in order to operate, nor does it require specific hardware devices; these two factors give our team an advantage in the arena of scalability of our deployments. XAHIVE will interface with hospital information systems at both sites—Canada and the United Kingdom—in order to realize (1) consistent security across all communication touch points in the SMArTVIEW system, and (2) a clear chain of custody on the privacy of data per legislative requirements. SMArTVIEW involves multiple “moving parts” that will generate data about recovering seniors’ status and behavior via peripheral devices. Additionally, the solutions we use will generate and aggregate clinical measurements in discrete locations. Third-party data sets (eg, health services utilization data) will also be accessed, allowing for correlations to be made beyond the scope of our integrated systems. There are multiple considerations in the way data is aggregated (eg, efficiency of architecture, reduction of redundancy, and optimization of data accessibility). As leading experts, mPath will govern our data aggregation practices.

### Scalability

With scalability central to our vision, all partner solutions are at technology readiness Level 9, with next to zero time to solution required. Our scalability report will include documentation of (1) unforeseen issues as they arise and problem solving strategies, (2) patient and SVN experience, and (3) results of our comprehensive econometrics evaluation plan, distilled into a projected model of total cost of 1-year SMArTVIEW patient throughput, based on site surgical volumes.

### Evaluation Plan Objectives

The objectives of the evaluation plan are to refine SMArTVIEW (Phase 1) and conduct an RCT to examine its effectiveness.

### Settings

Both evaluation plan phases will take place at Hamilton Health Sciences, Hamilton, Canada, and Liverpool Heart and Chest Hospital, the United Kingdom; the coordinating center is the Population Health Research Institute, Hamilton, Canada.

## Methods

### Phase 1: Usability Testing

#### Participants

Included participants will be (1) aged ≥65 years, (2) undergoing major CaVS with predicted admission >48 hours, and (3) able to read, speak, and understand English such that reflexive intervention surveys generated by eTrAC can be completed (ie, Grade 6 reading level). Those excluded will have planned postop admission or readmission to a nursing home or long-term care facility.

#### Design and Procedures

##### Overview

Rogers’ methods for usability testing [100] will guide SMArTVIEW refinement in the first 9 months. Patients and clinicians will engage in two cycles of focus groups and usability testing at each site, as described in the following sections.

##### Focus Groups

Two focus groups, one at each site, will each be conducted with 5 CaVS patients and 5 SVNs via an adapted semistructured interview guide [101]. With well-established technology partner solutions (ie, applications and devices), our focus is refining overall system intervention flow and staging. After viewing still images of each SMArTVIEW stage, feedback will be elicited about (1) what is seen in each still, (2) expectations for engaging with the SVN and eTrAC solutions at each stage, (3) what each stage should accomplish, and (4) if conceptualization of SMArTVIEW aligns with participants’ mental models of required tasks.

##### Usability Testing

High-fidelity user testing of SMArTVIEW, focused on intraoperability and flow of information, will involve a human factors analyst, a research assistant (RA) with design ethnography training, and the leadership team. Focus group findings will be embedded into test (ie, simulated) clinical scenarios, representing CaVS recovery issues. Using think-aloud [102,103] protocols and task completion checklists, this usability testing cycle—conducted twice, once at each site—will have SVNs and patients rehearse all scenarios wherein information coming from either player can be communicated, via automated monitoring or self-report. Scheduled for 2 hours in the hospital and 2 hours in the patient’s home soon after, but not on the same day, rehearsals will be observed by the human factors analyst and the RA. Through analysis of recorded usability testing data to identify patterns of use, areas of satisfaction or frustration, and system efficiencies and problems, the human factors analyst and the RA will determine actions for system refinement [104,105]. The entire cycle will be repeated, this time observing real-time-based interaction.

### Phase 2: Randomized Controlled Trial

#### Methods

##### Trial Design

**Figure 3:**
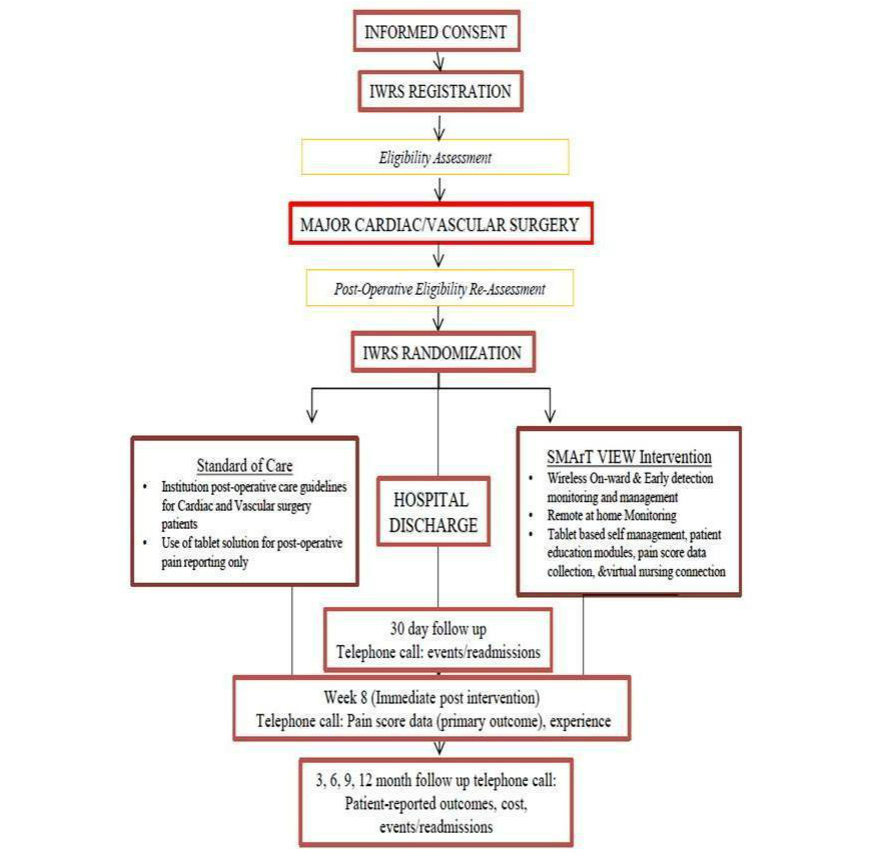
Randomized controlled trial flow diagram. IWRS: Interactive Web Randomization System.

Research questions to be addressed in a two-group, parallel-arm RCT (see Figure 3) include effectiveness of the intervention to (1) improve postop pain at 30 days (primary outcome) and (2) composite of major postop complications related to hemodynamic compromise, HQRL, depressive symptoms, health service utilization costs, and patient-level cost of recovery (secondary outcomes).

##### Participants

Participants will be included according to the inclusion/exclusion criteria outlined in Phase 1, with two additional exclusion criteria: (1) participation in Phase 1 and (2) positive Confusion Assessment Method (CAM) screening upon transfer to the surgical ward.

##### The Self-Management—Vision for Patient Empowerment Intervention

The components of the SMArTVIEW intervention will be as described under the Intervention Program, Technologies, and Effectiveness section. The intervention delivery protocol, by stage, is presented in Table 2. This protocol features SVN support from the surgical ward 24 hours/day, 7 days/week.

**Table 2:** SMArTVIEW^a^ intervention delivery protocol by stage.

Stages		Details
**Stage 1: In hospital**		
	**IntelliVue Guardian setup** **(ward day 1)**	
		Upon transfer to the ward, the IntelliVue Guardian early warning system is established by the SVN^b^ on duty, who connects patient to peripheral, cableless devices; establishes baseline/normal vital signs with spot check monitor; activates IntelliVue Guardian; and performs system checks every shift.
		The SVN will receive alerts via mobile device; alerts will be set according to surgeon-sanctioned vital signs parameters programmed into IntelliVue Guardian, which allow for tailoring of profiles for day or night, as well as pre-existing comorbid conditions (eg, atrial fibrillation).
		Upon alerts, SVN assessment, intervention, and escalation of care will be according to usual hospital protocols.
	**Patient and family pain education and hospital-to-home orientation** **(ward day 2)**	
		The SVN will facilitate a 2.5-hour hospital-to-home orientation session implemented at the convenience of the patient, supports (eg, family, friends, and caregivers), and clinical workflow.
		This orientation will focus on the eTrAC^c^ tablet-based applications, the PRAS^d^ educational video, and Restore.
		Following the orientation, the SVN will invite and answer questions.
	**Receipt of SMArTVIEW hospital-to-home package and skills rehearsal** **(day prior to discharge**)	
		On the day prior to discharge, patients will receive their hospital-to-home packages from the SVN, including eTrAC tablet-based solutions, instructions for monitoring vital signs at home, eTrAC 30-day application schedule for monitoring vital signs, SVN video visits, and daily recovery symptom and reflexive surveys.
		Upon receipt of this hospital-to-home package, the SVN will facilitate a 30-minute checklist-oriented rehearsal of all eTrAC features; the SVN will also invite and answer questions.
**Stage 2: In the community**		
	**Setup (week 1 postdischarge)**	
		Philips’ in-home installation team will work with the SVN to establish the Bluetooth-enabled vital signs monitoring system.
		The SVN will then commence monitoring of all incoming data from eTrAC via eCC^e^.
	**Patient monitoring and virtual check-ins** **(first 30 days postdischarge)**	
		The SVN will perform daily 15-minute virtual check-ins—eTrAC video visits—with patients at home from the hospital via eCC, per hospital-to-home package instructions.
		Virtual check-ins will include review of priorities flagged in eCC, review of vital signs and symptom and reflexive survey data, postop pain assessment, and discussion of any patient/SVN concerns.
		Issues identified—via eCC risk stratification or SVN assessment—that require intervention, but are out of the scope of SVN practice, will be escalated to the most responsible clinician.
	**SMArTVIEW-Restore** **(0 to 7 weeks postdischarge)**	
		During recovery, participants will engage the Restore time-release, self-guided, online curriculum (described previously).
		Restore is structured according to seven weekly asynchronous modules, consisting of two to seven activities each.
		Restore is designed to constitute 2-3 hours of online activity, weekly.

^a^SMArTVIEW: Self-MAnagemenT—VIsion for patient EmpoWerment.

^b^SVN: SMArTVIEW Nurse.

^c^eTrAC: Transition to Ambulatory Care.

^d^PRAS: pain relief after surgery.

^e^eCC: eCare Coordinator.

##### Outcome Measures

###### Primary Outcome

The primary outcome is *worst* postop pain intensity upon movement in the previous 24 hours—at 30 days after randomization—averaged across the previous 14 days. This will be assessed using the Brief Pain Inventory-Short Form (BPI-SF), which has well-established reliability and validity in surgical groups, including CaVS [15,106,107]. Common to studies with postop pain as a primary outcome [15,106,107], patients will report *worst* pain-intensity rating both upon rest and movement in the past 24 hours. The primary outcome of worst pain *upon movement* is a more reliable indicator of suboptimal pain management and pain-related interference with recovery-related activities than worst pain *upon rest* [15,106,107].

###### Secondary Outcomes

####### Postoperative Complications Related to Hemodynamic Compromise

We will capture a composite of complications related to hemodynamic compromise up to 30 days postrandomization, including death, myocardial infarction, and nonfatal stroke. The number of events for the overall composite, as well as number of events per component within the composite, will be reported.

####### All-Cause Mortality and Other Postoperative Complications

All-cause mortality will be captured up to 1 year postrandomization. We will also monitor for new-onset atrial fibrillation and SSI up to 30 days postrandomization.

####### Functional Status

The Short-Form 12 version 2 (SF-12v2) is an established, reliable, and valid tool [108,109] to measure functional status [108,109]. The SF-12v2 provides both physical component summary and mental component summary scores [9,11,46,109].

####### Depressive Symptoms

The five-question version of the Geriatric Depression Scale (GDS-5) will be used to measure depressive symptoms. This tool is a well-validated instrument in the assessment of depression in hospitalized older adults, with high levels of sensitivity and specificity [110,111].

####### Chronic Postsurgical Pain

Development of CPSP is defined [112] as (1) pain that developed after the surgical procedure, (2) being different from pain experienced before surgery, and (3) being present for at least 3 months. Patient responses in the affirmative to each of these questions indicate patients have developed CPSP. For patients who have developed CPSP, pain intensity and related interference with usual daily activities will be measured via the BPI-SF [106,107].

####### Heath Service Utilization-Related Cost

Data on hospital readmission and health care utilization and costs of health service utilization data from the Canadian arm of the trial will be linked with the health administrative Institute for Clinical Evaluative Sciences data repository. Administrative databases used to describe the health service utilization include (1) Registered Persons Database—demographics and vital statistics of all legal residents of Ontario, (2) Discharge Abstract Database—records of inpatient hospitalizations—from the Canadian Institute for Health Information (CIHI), (4) Ontario Health Insurance Plan Database—physician billing claims, and (5) the National Ambulatory Care Reporting System—information on emergency department visits—from CIHI. In addition, to capture data on times spent on the portal by health providers (eg, pharmacists and nurses), costs of health providers’ time will be captured in the system reporting. Costs of health providers’ time on the portal will be calculated by multiplying the time with unit costs from standard costing sources in Ontario.

####### Patient-Level Cost of Recovery

The Ambulatory and Home Care Record (AHCR) [11,113-117] will be used to comprehensively measure patient-level cost of illness from a societal perspective (Canada and the United Kingdom). This approach gives equal consideration to health system costs and costs borne by patients and unpaid caregivers (eg, family members and friends). AHCR items can be categorized as publicly financed (eg, public sector paid resources) or privately financed care (eg, all out-of-pocket and third-party insurance payments, and time costs incurred by caregiver). Face validity and reliability of the AHCR is well-established in multiple groups, including CaVS patients [11,113-117].

##### Baseline Measures to Inform Subgroup Analyses

Aside from baseline clinical and demographic information, gender-based pain expectations [118] will be assessed to inform subgroup analyses as evidence suggests that gender-based pain expectations may lead to differences in the experience of pain and related response to interventions [118]. These expectations will be captured using the Gender Role Expectations of Pain (GREP) tool, which captures stereotypic attributions regarding pain endurance, pain sensitivity, and willingness to report pain. The GREP tool has been used in multiple pain investigations [118-123] with acceptable test-retest and internal consistency reliability [118].

Baseline digital literacy will also be assessed using an adapted version of the informational and instrumental support domains of the Patient-Reported Outcomes Measurement Information System (PROMIS) measures. This approach, previously pilot-tested with cardiovascular patients [124], employs five items to examine current level of engagement with mobile and digital technologies.

##### Follow-Up

The SVN will collect outcome data for intervention and control groups following random allocation through discharge. Once in the community, patients in both groups will record their BPI-SF pain scores daily for 8 weeks using the tablet-based solutions. Data on 30-day event rates (ie, major postop complications) and hospital readmissions for both groups will be collected by a blinded RA via telephone interview at 30 days postoperation. At 3, 6, 9, and 12 months, additional telephone interviews conducted by the RA will assess (1) functional status, (2) depressive symptoms, (3) CPSP, and (4) patient-level cost of recovery (ie, AHCR).

##### Qualitative Data Collection

To understand patient experience with CaVS recovery and involvement with the SMArTVIEW intervention, we will conduct telephone interviews with 60 patients and 60 primary support persons in the intervention and control groups and with all SVNs (n=20) using a semistructured interview guide. The interviews will focus on perceptions of usability and ethical, social, and legal issues. Our sample size should ensure data saturation [125].

##### Sample Size

Assuming a two-sided type I error (alpha) of .05 and a standard deviation of 20 points in BPI-SF numeric rating scale scores (range 0-100), a total of 128 participants (ie, 64 individuals in each group) are required to provide 80% power to detect a minimally important difference of 10 points. This difference represents a moderate effect size (Cohen’s d=0.50) [126]. Assuming a 10% loss to follow-up, 144 total patients (or 72 per group) is required. We will use this sample size for each site, to allow for site-specific analyses with equal and sufficient power. If there is sufficient homogeneity between the Canadian and UK samples, the combined sample (eg, 256 patients with complete data) would provide 80% power to detect a difference in pain intensity scores of 8.6 points (Cohen’s d=0.43), assuming a generous design effect [127] of 1.5 due to the clustering of participants within the site.

##### Recruitment

Strategies previously developed will be applied [3,6]; RAs will screen preoperative surgical patient lists daily. Anesthesia, CaVS, and medicine services will contact the RAs for all CaVS admissions through emergency and new consultations. Eligible patients will be approached and invited. Patients providing informed consent will be registered via the Interactive Web Randomization System (IWRS), a 24-hour, central, computerized, secure (ie, password-protected), Web-based registration/randomization service at the Population Health Research Institute, and baseline data will be collected. Patients undergoing urgent surgeries will be approached postoperation.

##### Randomization and Allocation

Blocked randomization (ie, randomly assigned block sizes) will be used to achieve balanced allocation of intervention and control groups. The randomization allocation list will be prepared by Population Health Research Institute statisticians and integrated into the IWRS system. Upon transfer to the surgical ward (ie, post-ICU), the SVN will assess consented patients using the CAM. If CAM scores do not indicate cognitive impairment or delirium and the patient remains eligible, they will be randomly allocated by the IWRS.

#### Feasibility

Our technology partners have contributed equipment and personnel time, in-kind, such that we are able to intervene and follow up on 15 patients at one time per site throughout the study until 30 days follow-up, at which time equipment will be returned to each hospital site for cleaning and reset. Therefore, the RCT (Phase 2) will be executed in five serial, parallel waves of approximately 30 patients per site. In 2014, there were 2311 and 1974 CaVS performed at Hamilton Health Sciences, Canada, and the Liverpool Heart and Chest Hospital, the United Kingdom, respectively. Planned recruitment will occur at a rate feasible for SVN time and access to the surgical populations at both sites. Patients will be enrolled during a 3-week period, with the last week of each recruitment month available for additional patient registrations as needed to accommodate those not meeting postop eligibility. Each site will target recruitment of 14 cardiac and 16 vascular patients during 3 weeks of recruitment—10 patients per week, per site. Allowing for a 25% refusal rate, lost opportunities, and competing studies, this still provides access to 20 patients at Hamilton Health Sciences and 17 patients at the Liverpool Heart and Chest Hospital. Since participant recruitment is only limited by prototype availability, our proposed recruitment target and timeline is feasible and recruitment of 300 participants will be completed in 10 months’ time.

### Data Analyses

#### Primary Analyses

Table 3 summarizes all data analyses. Using the intent-to-treat principle [128], all patients will be included in the final analysis and according to the group to which they were randomly allocated. Descriptive statistics will be used to describe sample characteristics using measures of central tendency and dispersion for continuous factors, and frequencies and proportions for categorical factors. A two-sided significance level of .05 will be used for all inferential analyses. Statistical methods used will depend on the type and distribution of data for the outcome variable under study. If outcome data meet requirements for parametric statistics, a linear mixed model [129] will be used to assess the effects of the intervention on the primary outcome. An a priori contrast of the weekly average worst score for the BPI-SF numeric rating scale *upon movement* (previous 24 hours) will be used to evaluate the primary endpoint of acute postop pain at 30 days postrandomization.

Linear mixed models, using an autoregressive [130] covariance structure—allowing for correlations between measurements to decline as they are further apart in time—will be used to evaluate within-patient variation in patient-reported outcomes over 12 months of follow-up. Linear mixed models are a flexible and powerful approach to the analysis of data with a complex variance structure, such as correlated data [129-131]. Unlike traditional repeated-measures designs, these models do not require complete data on each patient and have increased statistical power [132]. Nonlinear mixed models will be used in the following cases: (1) if continuous data violate assumptions of normality [132] and (2) for categorical secondary outcomes (eg, adverse event). Chi-square tests of association will be used to assess the association between categorical secondary outcomes identified in the administrative data and intervention. Given that the data is derived from an RCT, complex modeling for these outcomes will not be performed, as potential confounders are considered to be adjusted for in the design. Finally, we will examine patterns of missing data and determine demographic and/or clinical characteristics that are related to missing data at each time point, and the potential impact on the primary findings.

#### Secondary Analysis

A secondary analysis will aim to establish the cumulative impact of the components of the intervention (eg, remote monitoring and self-management training) on outcomes and assess “digital retention” and sustained digital device usage in visual and time-sensitive analyses using an N-of-1 design (see Table 3). N-of-1 designs use a patient as their own control and can assess the impact of incremental changes with respect to the intervention with frequent and repeated measurements of the outcome variable of interest (ie, pain over time) and are particularly applicable to digital health and mobile phone-based clinical trials [133]. An N-of-1 design allows for association of causality to interventions in real time and direct methods to estimate individual treatment effects and variation per patient. Using the funnel approach, an individual patient is observed repeatedly to graphically demonstrate the variation in pain and HRQL over time [134].

#### Subgroup Analyses

Two types of separate subgroup analyses are planned to determine the impact of gender-based pain expectations and patient sex on intervention effectiveness (see Table 3):

1. Patients will be stratified into high versus low GREP scores. The primary analyses examining the effect of the intervention on the worst score for the BPI-SF numeric rating scale *upon movement* (previous 24 hours) will be conducted. An interaction term for GREP score (low versus high) and the group allocation will be incorporated into the analyses to determine if gender-based pain expectations are associated with differences in the effect of the intervention on the primary outcome. If a significant interaction is identified, the primary analysis in these two groups will be performed.

2. Similarly, interaction between the intervention and patient sex will be examined.

#### Cost-Effectiveness Analyses

The cost-effectiveness of implementing the intervention will be determined from two perspectives: (1) the Ministry of Health and Long-Term Care (MoHLTC) (Canada) and (2) society (Canada and the United Kingdom) (see Table 3). Separate analyses will be conducted from each perspective. MoHLTC costs will include costs associated with health service utilization over the study period (eg, hospitalization, emergency room visits, day surgery or procedure, laboratory services, outpatient visits, prescription drugs, and home care services from the Institute of Clinical Evaluative Sciences). Time that health providers (eg, pharmacists) spent on the SMArTVIEW portal will be calculated by multiplying the time with unit costs from standard costing sources in Ontario. From the societal perspective, costs will include those from the MoHLTC perspective, including costs incurred to patients and family members (eg, travel cost and productivity loss), which will be captured through the AHCR.

The first economic analysis outcome is the incremental cost of the intervention compared to usual care. We will analyze the total cost as a dependent variable, using a regression model to estimate the difference in expected health care cost between the two groups. The intervention will be the primary independent variable and the regression model will adjust for potential confounding variables. In theory, an ordinary least squares model produces unbiased estimates even if the data are skewed [135,136]; however, additional estimation methods (eg, generalized linear models) and different uncertainty methods (eg, parametric and nonparametric bootstrapping) will be explored to facilitate investigation of the impact of various cost assumptions.

As a secondary cost objective, we will compare the cost and quality-adjusted life years (QALYs) between the two groups using the net benefit regression framework (see Table 3). QALY is a preference-based utility measure of HRQL, as perceived by the patient, that incorporates both length of life and quality of life into a single measure [137,138]. We aim to determine the incremental net benefit of interventions versus usual care. To estimate QALYs gained, we will convert SF-12v2 data collected to utility scores using a validated algorithm. We will also estimate the incremental cost per QALY gained. A cost-effectiveness acceptability curve (95% CI) will be used to characterize the uncertainty of our findings [139].

#### Uptake of Technology

Data on uptake of the SMArTVIEW intervention will be used to explain differences in the outcome measures, determine patterns of use to predict outcomes, and identify users who may require escalated care. Session frequency (ie, times the technology is accessed) and session length (ie, length of time users interact with the technology) [140] will be determined via daily metrics of both device and application use. During Phase 1, mPath will identify all key actions of SMArTVIEW to generate a template for relevant data collection at a granular, individual user level.

**Table 3:** Summary of outcomes, hypotheses, measures, and methods of analysis.

Analyses	Outcome	Hypothesis	Outcome measure	Method of analysis
**Primary outcome**				
	8-week worst postop pain intensity *upon movement* score in the past 24 hours	Intervention > control	Measured by Brief Pain Inventory-Short Form (BPI-SF^a^)	Linear mixed model or nonlinear mixed models (if assumptions of normality are violated)
**Secondary outcome**				
	Functional status	Intervention > control	Short-Form 12 version 2	Linear mixed model or nonlinear mixed models (if assumptions of normality are violated)
	Depressive symptom scores	Intervention > control	Five-question version of the Geriatric Depression Scale	Linear mixed model or nonlinear mixed models (if assumptions of normality are violated)
	Postop complications related to hemodynamic compromise	Intervention > control	Myocardial infarction and stroke	Nonlinear mixed models
	Other relevant postop complications	Intervention > control	Surgical site infection, presence of CPSP^b^	Nonlinear mixed models
	Heath service utilization-related cost	Intervention > control	Linked with health administrative Institute for Clinical Evaluative Sciences data repository	Linear mixed model or nonlinear mixed models (if assumptions of normality are violated)
	Patient-level cost of recovery	Intervention > control	Ambulatory and Home Care Record	Linear mixed model or nonlinear mixed models (if assumptions of normality are violated)
**Subgroup outcomes**				
	All outcomes	Effect will differ by gender (male versus female)	Worst postop pain intensity *upon movement* score in the past 24 hours measured by BPI-SF numeric rating scale	Interaction test
	All outcomes	Effect will differ by GREP^c^ scores (low versus high)	Worst postop pain intensity *upon movement* score in the past 24 hours measured by BPI-SF numeric rating scale	Interaction test

^a^BPI-SF: Brief Pain Inventory-Short Form.

^b^CPSP: chronic postsurgical pain.

^c^GREP: Gender Role Expectations of Pain.

### Qualitative Analyses

#### Qualitative Description

Data will be digitally recorded, transcribed verbatim, and managed in NVivo 11 (QSR International). Concepts that relate to the usability and value of the intervention will be coded [141] and reviewed by investigators to resolve differences and minimize biases [141]. Revisions to the interview guide and codebook will reflect emerging themes.

#### Critical Qualitative Analysis

To reveal the ethical, legal, and social implications of the intervention, we will apply methods used successfully in previous research [142]. Following this, data will be re-examined using four bioethical concepts—relational autonomy, care, social justice, and privacy—to draw out the normative implications of the intervention which are sensitive to ethical issues common to at-home care for seniors [143]. A *retroductive* process will be used that involves moving between observations and concepts and allows the interplay between individuals’ lives and larger social and contextual forces to be understood [144,145]. The four concepts will not be used simply as containers to categorize data uncritically to aid in the social and ethical analysis of the data. Rigor will be maintained by keeping a reflexive journal and audit trail [141] and ensuring that the purpose of the research, theoretical assumptions, and method of data analysis are congruent [146].

### Controls for Bias

To limit sampling bias, a recruitment schedule randomly generated to ensure representation from each surgical group will be used. Contamination should not exist between groups as we will control who interacts with the SVN and intervention features. Those allocated to the intervention group will be asked not to share their tablets or demonstrate application features to any peers assigned to the control group. To evaluate cointervention, we will track participant receipt of any monitoring or recovery support-related interventions, outside of expected usual care up to 8 weeks postoperation. RAs responsible for outcome data collection will be separated from randomization procedures, will have no permitted access to IWRS, will not be involved in intervention delivery, and will be blinded to group allocation. An event adjudication committee responsible for adjudication of all clinical outcome data will be blinded to randomized allocation. The team has extensive experience with assiduous follow-up procedures to minimize losses to follow-up.

### Knowledge Translation

Integrated knowledge translation strategies will continue to involve stakeholder groups during the project. As part of integrated and end-of-grant knowledge translation, stakeholders will assist in interpreting findings, identifying key results, and reviewing and revising the end-of-grant knowledge translation plan at a final meeting for review of investigator results. End-of-grant knowledge translation goals—generate interest, discussion, and awareness; impart knowledge; and inform research—will be addressed via tailored implementation strategies.

## Results

Study start-up is underway and usability testing is scheduled to begin in the fall of 2016.

## Discussion

THE SMArTVIEW, CoVeRed innovation community brings together an international, dedicated group of well-known clinical and eHealth researchers; health economists; clinicians; administrators; patient representatives; engineers, information technology, and clinical informatics experts; as well as leaders in the arenas of health policy, big data and data aggregation, bioethics, knowledge translation, and privacy. Collectively, we possess the requisite skills, experience, and track record to execute the proposed evaluation, disseminate what we learn, and plan for diffusion of innovation.

We are actively engaged in systems integration and change management at both study sites. As a result of planned, shared stewardship of our vision, we have fostered a milieu of co-ownership and investment in SMArTVIEW usability and effectiveness testing. With respect to end-user engagement, we understand well from experience that innovation is not a linear process. We are committed to recursive coinnovation between “solutioner” and end users. Hence, corefinement of SMArTVIEW— via usability testing in context—was a key objective identified during team debriefing, following our patient journey mapping exercise. Akin to the use of “experimentation suites” in the industry sector, our process, as outlined within the Usability Testing section, will be to immerse with participants in high-fidelity rehearsal of SMArTVIEW activities in order to uncover ways we can refine our processes to optimize the experience of recovery for seniors following CaVS. Our team, including patient representatives, is organized into both content and governance committees (see Table 4).

In collaboration with our industry partners, Canadian and UK hospital sites, we are confident that we have the experience, expertise, infrastructure, and support to realize THE SMArTVIEW, CoVeRed. A copy of our Canadian Institutes of Health Research (CIHR) reviews can be found in Multimedia Appendix 2.

**Table 4:** SMArTVIEW^a^ team committees.

Committee type	Committee name
**Content**	
	Clinical Transformation/Change Management
	Clinical Monitoring
	Patient Engagement and Experience
	Economics
	Knowledge Translation
	Systems Integration
	Self-Management
	Clinician and SVN^b^ Training
	Ethics
**Governance**	
	Project Office Operations
	International Operations
	Steering
	Outcomes Adjudication
	External Safety
	Efficacy and Monitoring
	Substudy and Publications

^a^SMArTVIEW: Self-MAnagemenT—VIsion for patient EmpoWerment.

^b^SVN: SMarTVIEW Nurse.

## Appendix

Multimedia Appendix 1 Approach to meta-analysis.

Multimedia Appendix 2 Canadian Institutes of Health Research (CIHR) reviews.

## Abbreviations

AHCR: Ambulatory Home Care Record
BPI-SF: Brief Pain Inventory-Short Form
CAM: Confusion Assessment Method
CARD: Cardiac
CaVS: cardiac and vascular surgery
CIHI: Canadian Institute for Health Information
CIHR: Canadian Institutes of Health Research
CPSP: chronic postsurgical pain
eCC: eCare Coordinator
ECG: electrocardiogram
eTrAC: Transition to Ambulatory Care
EWS: early warning score
GDS-5: five-question version of the Geriatric Depression Scale
GREP: Gender Role Expectations of Pain
HOPE: Help to Overcome Problems Effectively
HR: hazard ratio
HRQL: health-related quality of life
ICU: intensive care unit
iHOPE: Internet-based Help to Overcome Problems Effectively
IWRS: Interactive Web Randomization System
MoHLTC: Ministry of Health and Long-Term Care
N/A: not applicable
PCI: percutaneous coronary intervention
POISE: PeriOperative ISchemic Evaluation
postop: postoperative
PRAS: pain relief after surgery
PROMIS: Patient-Reported Outcomes Measurement Information System
QALY: quality-adjusted life year
RA: research assistant
RCT: randomized controlled trial
SF-12v2: Short-Form 12 version 2
SpO2: peripheral oxygen saturation
SSI: surgical site infection
SVN: SMArTVIEW Nurse
THE SMArTVIEW, CoVeRed: TecHnology-Enabled remote monitoring and Self-MAnagemenT—VIsion for patient EmpoWerment following Cardiac and VasculaR surgery
