# Supplementary material for: Technology-Enabled Remote Monitoring and Self-Management — Vision for Patient Empowerment Following Cardiac and Vascular Surgery: User Testing and Randomized Controlled Trial Protocol
Source: JMIR Res Protoc. 2016 Aug 1;5(3):e149. doi: 10.2196/resprot.5763 (PMC4999307; doi:10.2196/resprot.5763)
Supplement: Multimedia Appendix 1 [file resprot_v5i3e149_app1.pdf]

Approach to Meta-Analysis

Search for identification of studies

We searched the Cochrane Library, MEDLINE, PubMed, CINHAL, EMBASE, Proquest Dissertation Abstracts, and Psychinfo, between Jan. 1990 – Dec. 2014, using combinations of key medical subject heading (MeSH) terms including ‘cardiac surgery’, ‘vascular surgery’, ‘chronic post-surgical pain’, ‘persistent post-surgical pain’ and ‘acute postoperative pain’. We also conducted hand searches of relevant journals and secondary references, as well as proceedings of international conferences; experts were also consulted for additional sources. Restriction was applied to English language only.

Final selection of Studies

Four reviewers (MM, SH, SC, JY) reached consensus on relevant articles to be included in this analysis by reviewing the titles, abstracts and results of studies according to the inclusion criteria.

Data extraction

Four reviewers (MM, SH, SC, JY) participated in independent quality assessment and extraction of process and outcome data from each study according to a standardized extraction format we have used in other reviews.

Data synthesis and analysis

Standardized mean differences and 95% confidence intervals were used to allow the pooling of effect sizes from studies using either dichotomous (presence of pain immediately post-op) or continuous pain scores (e.g., NRS) as predictors of chronic post-surgical pain. Standardized mean differences were calculated using RevMan 5.1.7® software. Standardized mean differences were determined using odds ratios or risk difference in the probability of chronic postsurgical pain between those who did and did not develop chronic post-surgical pain, or by calculating the standardized mean difference in acute pain scores between those who did and did not develop chronic post-surgical pain. A standardized mean difference of 0.20 standard deviation units was considered a small difference between outcome groups, while 0.50 was a moderate difference, and 0.80 represented a large difference. We used standard inverse-variance, random-effects meta-analysis to combine the studies.
