# Supplementary material for: Technology-Enabled Remote Monitoring and Self-Management — Vision for Patient Empowerment Following Cardiac and Vascular Surgery: User Testing and Randomized Controlled Trial Protocol
Source: JMIR Res Protoc. 2016 Aug 1;5(3):e149. doi: 10.2196/resprot.5763 (PMC4999307; doi:10.2196/resprot.5763)
Supplement: Multimedia Appendix 2 [file resprot_v5i3e149_app2.pdf]

**Review Type/Type d'évaluation:** Committee Member 1/Membre de comité 1
**Name of Applicant/Nom du chercheur:** MCGILLION, Michael Hugh
**Application No./Numéro de demande:** 348440
**Agency/Agence:** CIHR/IRSC
**Competition/Concours:** 2015-06-03 Operating Grant: eHealth Innovations Initiative: eHealth Innovation Partnership Program (eHIPP)/Subvention de fonctionnement: Initiative Innovations en cybersanté : Programme de partenariats pour l'innovation en cybersanté (PPIC)
**Committee/Comité:** eHealth Innovation Partnership Program (eHIPP): Support of Seniors/Programme de partenairiats pour l'innovation en cybersanté-L'appui awx personnes âgéess
**Title/Titre:** THE SMArT VIEW, CoVeRed: TecHnology Enabled monitoring and Self-MAnagemenT- VIsion for patient EmpoWerment following Cardiac and VasculaR surgery

---

**Assessment/Évaluation:**

**Brief synopsis**

This project proposes to test the effectiveness of the use of SMArt VIEW, which monitors BP, respiration, $SpO_2$ (concentration of oxygen in the blood), temperature, and weight to prevent hemodynamic compromise (blood flow disturbance, e.g. hypotension leading to heart attack) for patients post cardiac or vascular surgery. They will use the device in the hospital and at home for 8 weeks. At home, they will use the Phyllip monitoring system, send vital signs, report pain level and wound photo, as well as video conferencing with the nurse. They will test usability in the 1$^{st}$ phase and in the 2$^{nd}$ phase, they will conduct a RCT using 128 Canadian patients, and half (n=64) will use the device for 8 weeks post-surgery. They will follow up the patients at 3, 6, 9, and 12 months. The primary outcome is pain (due to increased sensitization to pain), the secondary outcomes are quality of life, depression, complications, and health service utilization costs. At 8 weeks they will also conduct phone surveys with 60 patients and 60 caregivers for experience of recovery. The test will be conducted in both Canada and UK, which have different health systems, because their product, the SMArt VIEW, has been jointly developed and invested.

**Comments**

Over all, this is a very strong proposal, which addresses the need of intervention for at risk population. Uniqueness was added due to the involvement of UK due to the joint development of the concept, technology, and investment. More developed scale-up plan will strengthen this project.

**Planned Intervention:**

**<u>Strength</u>**

1. This proposal deals with high-risk patients for whom the intervention is a must to prevent serious adverse events due to severity and complexity of the disease as well as surgical site infections.
2. The project is based on thorough research of past projects and results.
3. Technology, Phillip's Intelli Vue Guadiana (IG) collects data and notifies an early warning score for deterioration to the hospital, which was conventionally done manually, and to the nurse' mobile device. The strength is this includes patient education (2-hour/week X 6 weeks) about self-management, especially pain, after the surgery. The device was tested with other types of patients and showed 45% reduced hospitalization.
4. A long term follow up plan strengthen the evidence.
5. "The right information is provided to the right person, at the right time concept" is clearly demonstrated by the Figure 6.

**Review Type/Type d'évaluation:** Committee Member 1/Membre de comité 1
**Name of Applicant/Nom du chercheur:** MCGILLION, Michael Hugh
**Application No./Numéro de demande:** 348440
**Agency/Agence:** CIHR/IRSC
**Competition/Concours:** 2015-06-03 Operating Grant: eHealth Innovations Initiative: eHealth Innovation Partnership Program (eHIPP)/Subvention de fonctionnement: Initiative Innovations en cybersanté : Programme de partenariats pour l'innovation en cybersanté (PPIC)
**Committee/Comité:** eHealth Innovation Partnership Program (eHIPP): Support of Seniors/Programme de partenairiats pour l'innovation en cybersanté-L'appui awx personnes âgéess
**Title/Titre:** THE SMArT VIEW, CoVeRed: TecHnology Enabled monitoring and Self-MAnagemenT- VIsion for patient EmpoWerment following Cardiac and VasculaR surgery

---

**Assessment/Évaluation:**

6. Technology Readiness level is 9.

-

**Weakness**

1. The scalability plan to province and regions is not solid, although technology by *XAHIVE* itself has built-in scale-up capacity due to no requirement services and no specific hardware devices (off the shelf).
2. For the RCT, only one hospital with a small number of participants (n=128) in Canada will be involved.

**eHealth Innovation Evaluation:**

**Strength**

Most approaches make sense.

**Weakness**

The sample size calculation should be based on a previous study and clarify what was the dependent variable in this process. It is assuming a medium effect size but where did it come from? Also what is the reason that two-sided test is used for the calculation? The previous study showed that the device use, then one-sided test can be used, leading to a smaller sample size.

1. The use of single subject method (N-of-1 design) has a generalization problem. Do you conduct 128 single subject method analyses and somehow draw a conclusion?
2. How the total cost can be a dependent variable for a regression needs a further explanation.

**Strength**

1. The PIs are well qualified for this project. Technology is well advanced, appropriate for this project, and easy scalability is attractive.

Weakness

1. With two countries involved in this project, as well as such a large number of people participating in this project, the coordination and communication methods should be clearly stated. This is not in review criteria.
2. Scale up plan in Ontario is not discussed.

| | |
|---|---|
| **Review Type/Type d'évaluation:** | Committee Member 1/Membre de comité 1 |
| **Name of Applicant/Nom du chercheur:** | MCGILLION, Michael Hugh |
| **Application No./Numéro de demande:** | 348440 |
| **Agency/Agence:** | CIHR/IRSC |
| **Competition/Concours:** | 2015-06-03 Operating Grant: eHealth Innovations Initiative: eHealth Innovation Partnership Program (eHIPP)/Subvention de fonctionnement: Initiative Innovations en cybersanté : Programme de partenariats pour l'innovation en cybersanté (PPIC) |
| **Committee/Comité:** | eHealth Innovation Partnership Program (eHIPP): Support of Seniors/Programme de partenairiats pour l'innovation en cybersanté-L'appui awx personnes âgéess |
| **Title/Titre:** | THE SMArT VIEW, CoVeRed: TecHnology Enabled monitoring and Self-MAnagemenT- VIsion for patient EmpoWerment following Cardiac and VasculaR surgery |

**Assessment/Évaluation:**

| | |
|---|---|
| **Review Type/Type d'évaluation:** | Committee Member 2/Membre de comité 2 |
| **Name of Applicant/Nom du chercheur:** | MCGILLION, Michael Hugh |
| **Application No./Numéro de demande:** | 348440 |
| **Agency/Agence:** | CIHR/IRSC |
| **Competition/Concours:** | 2015-06-03 Operating Grant: eHealth Innovations Initiative: eHealth Innovation Partnership Program (eHIPP)/Subvention de fonctionnement: Initiative Innovations en cybersanté : Programme de partenariats pour l'innovation en cybersanté (PPIC) |
| **Committee/Comité:** | eHealth Innovation Partnership Program (eHIPP): Support of Seniors/Programme de partenairiats pour l'innovation en cybersanté-L'appui awx personnes âgéess |
| **Title/Titre:** | THE SMArT VIEW, CoVeRed: TecHnology Enabled monitoring and Self-MAnagemenT- VIsion for patient EmpoWerment following Cardiac and VasculaR surgery |

**Assessment/Évaluation:**

This exceptionally strong proposal crosses health care sectors, continents, and analytical methods with the goal of delivering better care and outcomes to individuals who have had cardiac and vascular surgeries. The patient population, setting, and technology readiness all meet the qualification requirements for this funding competition.

The team assembled – investigators, patient and clinical partners, and technology partners alike – is impressive and provides a rich and diverse set of skills and experiences to contribute to the project. From pre-project consensus-building to rich plans for knowledge translation, the proposal is well-defined and conceived. The support letters and extent of commitments assembled are impressive and in many cases build on established relationships/networks. The methods described for the evaluation are appropriate and should offer a rich mixed methods perspective on the results. The lay summary of the project was very well written.

Minor questions/suggestions that arose on reading the proposal include:

- Why would individuals who live in a nursing home/long term care facility be excluded from the intervention?
- Given the length of this study, has/will the investigator team identify a stopping rule based on interim analysis of results?
- How will the post-discharge component of the study connect with primary care providers to ensure continuity of care?

| | |
|---|---|
| **Review Type/Type d'évaluation:** | Committee Member 3/Membre de comité 3 |
| **Name of Applicant/Nom du chercheur:** | MCGILLION, Michael Hugh |
| **Application No./Numéro de demande:** | 348440 |
| **Agency/Agence:** | CIHR/IRSC |
| **Competition/Concours:** | 2015-06-03 Operating Grant: eHealth Innovations Initiative: eHealth Innovation Partnership Program (eHIPP)/Subvention de fonctionnement: Initiative Innovations en cybersanté : Programme de partenariats pour l'innovation en cybersanté (PPIC) |
| **Committee/Comité:** | eHealth Innovation Partnership Program (eHIPP): Support of Seniors/Programme de partenairiats pour l'innovation en cybersanté-L'appui awx personnes âgéess |
| **Title/Titre:** | THE SMArT VIEW, CoVeRed: TecHnology Enabled monitoring and Self-MAnagemenT- VIsion for patient EmpoWerment following Cardiac and VasculaR surgery |

**Assessment/Évaluation:**

Comments: This is an example of a strong research initiative designed to increase patient self management through technology assisted nursing care. As an international study it provides full use of an cultural ethnography of discharge support post surgery. The model is clearly outlined in many schematics and time tables but I struggled to detect how the budget and tasks outlined were connected to the international study itself. The strength of this model is the intensive use of participatory design in the form of focus group guided reactions to photos of the various phases of the SMArT VIEW intervention and the iterative enclosed rehearsals of nurse patient interactions according to a number of personas. This is a highly focused, intensive, collaborative research project which attempts to increase patient engagement in post surgical care. The use of nurse; patient pairs, committee members, ethnographers, technical specialists in the rehearsals and in roll out is a high cost but potentially powerful methodology.

Planned intervention: The potential for cost savings, patient safety and patient engagement for post surgical care with CaVS patients is very high in this study. Although it was not specifically stated, intervention is coordinated through a common personal tablet and changing sensors used by patients on the ward, step down hospital units and in the home. This enables the patient and family to become familiar with the technology in a supported environment before the installation in the home and maximizes the feeling of security as medical support is being reduced. The intervention includes responding to automated monitoring of vital signs and alerts in hospital and in home; 10 minute virtual check in using QoC home software recovery platform and virtual 2 hour online self management sessions in groups of 15 patients. It would be helpful to see how these interventions link to the larger study that this current application relates to.

| | |
|---|---|
| **Review Type/Type d'évaluation:** | Committee Member 3/Membre de comité 3 |
| **Name of Applicant/Nom du chercheur:** | MCGILLION, Michael Hugh |
| **Application No./Numéro de demande:** | 348440 |
| **Agency/Agence:** | CIHR/IRSC |
| **Competition/Concours:** | 2015-06-03 Operating Grant: eHealth Innovations Initiative: eHealth Innovation Partnership Program (eHIPP)/Subvention de fonctionnement: Initiative Innovations en cybersanté : Programme de partenariats pour l'innovation en cybersanté (PPIC) |
| **Committee/Comité:** | eHealth Innovation Partnership Program (eHIPP): Support of Seniors/Programme de partenairiats pour l'innovation en cybersanté-L'appui awx personnes âgéess |
| **Title/Titre:** | THE SMArT VIEW, CoVeRed: TecHnology Enabled monitoring and Self-MAnagemenT- VIsion for patient EmpoWerment following Cardiac and VasculaR surgery |

**Assessment/Évaluation:**

Innovation: The innovation for this particular study would seem to lie in the use of integrated software platforms for RPM with the addition of intensive participatory design and group weekly self management sessions. Of the entire study, I consider the online group sessions paramount as a way of facilitating social support for self management of care in a time of high stress and fear of being left alone to cope. With this important element, patients and families have a chance to be part of the research and thus understand the relevance of measured outcomes and interventions. I may be reading more into this than is intended but this project stands out in the use of technology to promote peer support.

Team: The team is well conceived, strong and inclusive. The existence of a UK partner opens the door to cultural differences in patient roles in health that may be informed by the UK base in patient engagement in health care. While patients are not part of the research team, they are fully engaged in the roll out of the technology.

| | |
|---|---|
| **Review Type/Type d'évaluation:** | Committee Member 4/Membre de comité 4 |
| **Name of Applicant/Nom du chercheur:** | MCGILLION, Michael Hugh |
| **Application No./Numéro de demande:** | 348440 |
| **Agency/Agence:** | CIHR/IRSC |
| **Competition/Concours:** | 2015-06-03 Operating Grant: eHealth Innovations Initiative: eHealth Innovation Partnership Program (eHIPP)/Subvention de fonctionnement: Initiative Innovations en cybersanté : Programme de partenariats pour l'innovation en cybersanté (PPIC) |
| **Committee/Comité:** | eHealth Innovation Partnership Program (eHIPP): Support of Seniors/Programme de partenairiats pour l'innovation en cybersanté-L'appui awx personnes âgéess |
| **Title/Titre:** | THE SMArT VIEW, CoVeRed: TecHnology Enabled monitoring and Self-MAnagemenT- VIsion for patient EmpoWerment following Cardiac and VasculaR surgery |

**Assessment/Évaluation:**

Tens of thousands of cardiac and vascular surgeries are performed on seniors every year in Canada and the UK. The current system of monitoring patients after an operation is manually checking at specified intervals which often get missed resulting missing or delaying detection in complications leading to stroke. The goal of the project is to implement sensors on patients and perform real-time data monitoring and reading for doctors to prevent missed readings and improve post operation outcomes.

The relevance of the population and inefficiencies are clearly defined. There are thousands of cardiac and vascular surgeries are done each year and given health epidemics this will continue to grow. The proposed technology is made up of components from multiple tech partners. Individually the proposed technology meets TRL requirement of 6-9 currently and are currently being sold in the market. The proposed solution would be an integration of multiple technologies and systems from Philips, QoC Health and XAHIVE. Meeting the TRL level of 6-9 within one year is a large goal given the size of the project and number of partners involved but it is achievable. Given the large number of cardiac problems associated with the priority area this innovation could reduce repeat visits and make the current investments being made have a higher return. The major costs are being provided in-kind for the purposes of the project. In order to fully scale this up and be actively deploying, significant costs would need to be covered and could be a barrier.

The environment is made up of two sites in relative close proximity to parties involved and given the support of the facilities is overall a good fit. Large major partners from all aspects are involved and on different levels have actively worked together before which allowed for this large group to be brought together. For the purposes of the proposal scale up should be adequate given the size of the technology partners. The technology itself does not have many barriers to scale given the cloud nature of it. The main scale barrier would be acquiring the hardware required however Philips is providing that in-kind. The management and leadership is strong and comprehensive with a broad scope making the overall team high quality. The facilities offered by those involved provide a complete and supporting environment. Two sites are available and the environment is all-encompassing for the requirements involved. Generally speaking all personnel, facilities and infrastructure are available and accessible. The exceptions are those personnel involved from the United Kingdom which may be harder to access given geographic location and time differences.

Overall the partner companies involved do have sufficient financial security. The major costs are being

**Review Type/Type d'évaluation:** Committee Member 4/Membre de comité 4
**Name of Applicant/Nom du chercheur:** MCGILLION, Michael Hugh
**Application No./Numéro de demande:** 348440
**Agency/Agence:** CIHR/IRSC
**Competition/Concours:** 2015-06-03 Operating Grant: eHealth Innovations Initiative: eHealth Innovation Partnership Program (eHIPP)/Subvention de fonctionnement: Initiative Innovations en cybersanté : Programme de partenariats pour l'innovation en cybersanté (PPIC)
**Committee/Comité:** eHealth Innovation Partnership Program (eHIPP): Support of Seniors/Programme de partenairiats pour l'innovation en cybersanté-L'appui awx personnes âgéess
**Title/Titre:** THE SMArT VIEW, CoVeRed: TecHnology Enabled monitoring and Self-MAnagemenT- VIsion for patient EmpoWerment following Cardiac and VasculaR surgery

---

**Assessment/Évaluation:**

covered by Phillips which is a multinational company. The other two companies involved have the infrastructure and businesses to a point where what they are being asked to provide is not overwhelming significant. The benefits of the technology are seen when all of the partners put their pieces together. On their own benefits exist but through a combination of them an integrated solution that can help make cardiac related procedures more successful in the long run and reduce hospital visits and health care costs is the main benefit. The technology solution itself is comprised of multiple items from multiple vendors not one particular unified solution as single end item. Commercialization beyond this project would require partnerships from all of the current technology partners and a coherent strategy and game plan. This may be difficult given the varying sizes and business maturity levels of the partners involved. Overall the capacity of the companies to commercialize is good. Phillips is providing off-the-shelf technology and nothing significantly new is required. The other companies are providing cloud software solutions that require minimal capacity to commercialize from their current position. The adoption potential of this technology where the project is being tested is very high assuming it provides positive results. Beyond the scope of this project the adoption potential of this technology is moderate at best. It is reduced outside of this environment because in order for adoption multiple companies need to be involved and coordinated which in itself is a large task. In addition, given the size of what would need to adopted (multiple pieces), there is often many bureaucratic and administrative barriers that would need to be overcome.

The budget seems to be applicable and nothing disproportionately stands out. Given the size of group, the parties involved the in-kind donations, support letters and related, it is within the scope of the project and this should be funded.
